# Daily Stress and Behavioral Problems in Chinese Children: The Moderating Roles of Family Functioning and the Classroom Environment

**DOI:** 10.3389/fpsyg.2021.742293

**Published:** 2021-10-27

**Authors:** Lili Wu, Fangyuan Ding, Tianqiang Hu, Gang Cheng, Xiaoyu Chen

**Affiliations:** ^1^Brain and Cognitive Neuroscience Research Center, Liaoning Normal University, Dalian, China; ^2^Faculty of Psychology, Southwest University, Chongqing, China; ^3^School of Psychology, Guizhou Normal University, Guiyang, China

**Keywords:** children, daily stress, behavioral problems, family functioning, classroom environment

## Abstract

Grounded in the stress-coping model, our study examined family functioning and the classroom environment as protective factors in the relationship between daily stress and behavioral problems in Chinese children. The participants were 1,450 children (51.7% male, *M*_*age*_ = 10.91 years, *SD* = 0.96) in the fourth, fifth, and sixth grades at five schools. The children completed the questionnaires measuring daily stress, family functioning, and the classroom environment. Additionally, their parents rated their behavioral problems. The latent moderated structural (LMS) equation approach was used to test moderator effects. After controlling for sex and grade, our results indicate that daily stress positively predicted the children’s behavioral problems. Both family functioning and the classroom environment moderated the relationship between daily stress and behavioral problems. Further assessment of latent interaction effects indicate that buffering effects on behavioral problems were most prominent in conditions involving low stress. In sum, families and schools should not ignore children’s minor stressors, as interventions involving family functioning and favorable classroom environments may help to reduce behavioral problems in children who report low levels of daily stress.

## Introduction

Daily stress—defined as “irritating, frustrating, distressing demands that to some degree characterize everyday transactions with the environment”—is increasingly recognized as an important risk factor for individual growth, with more daily stressors linked to behavioral problems (Schönfeld et al., 2016). Nevertheless, most studies have only considered domain-specific daily stress such as peer-linked, family-linked, and school-linked stress. Multiple facets of stress that may work synergistically are more potent than a single facet (Schneiderman et al., 2005), and children often face multiple, intersecting, cumulative stressors in their daily lives. They are likely to successfully manage isolated adverse events, but experience difficulty coping with ongoing stress.

In addition, some studies on daily stress and behavioral issues have been conducted with adolescents and adults, but research on children is limited (Rosenberg et al., 2016). Moreover, although social support may serve as a buffer against the impact of daily stress on behavioral problems, existing research on this matter has been conducted predominately in Western countries, with few studies carried out in China. It is unclear whether findings from Western nations are generalizable to Chinese individuals, who have different traditions and family structures. To fill this research gap, we aimed to investigate the relationship between overall daily stress and behavioral problems, and to identify contextual factors that may diminish this relationship in Chinese children.

Daily childhood stress can be classified into three main categories: (1) illnesses and events that involve a concern with body image; (2) stressful events in the academic context; and (3) negative events in family and school settings (Escobar et al., 2011). Daily childhood stress predicts emotional problems (i.e., anxiety and depression), behavioral challenges, and school maladjustment (Bai and Repetti, 2018). Moreover, daily stress predicts mental and physical well-being more strongly than infrequent major life events (Compas, 1987). In addition, the association between daily stress and mental health is stronger for children than for adolescents (Twenge, 2000).

Behavioral problems occur at all ages and often begin during childhood (Magai et al., 2018). Children in the fourth through sixth grades are on the threshold of adolescence, a time filled with transition and change (Rew et al., 2012). With increased participation in peer group activities, children’s social and emotional growth—that is, the ability to react to and interact with their social environment—undergoes a profound change. If children can develop prosocial relationships, obtain a sense of confidence, and express and manage their emotions, they are more likely to succeed in school. If not, a sense of inferiority can be particularly haunting during middle and late childhood (Raver, 2002). Research has indicated that behavioral problems are associated with poor-quality social relationships and learning difficulties (Chen et al., 2005).

Over the past 30 years, many epidemiological studies of childhood behavioral problems have been conducted in different countries. A recent meta-analysis estimated the worldwide prevalence of behavioral challenges in children to be 13.4% (Polanczyk et al., 2015). A review of child psychopathology studies found that the prevalence estimates of behavioral problems ranged from approximately 1% to nearly 51%, with a mean of 15.8% in Western countries (Roberts et al., 1998). A meta-analysis of Asian countries reported a general prevalence of 10–20% (Srinath et al., 2010). In China, children’s behavioral problems are serious, and have shown an escalating trend in recent years (Chinese Center for Disease Control and Prevention, 2020). Yang et al. (2019), through a field survey, found that among 9,295 Chinese students aged 6–16, the total detection rate of behavioral difficulties was 16.7%. If not managed properly, these problems could hinder children’s development and exert long-term negative influences, including subsequent antisocial behavior, active suicidal ideation, and poor academic performance (Crocetti et al., 2013; Kremer et al., 2016; Sarkisian et al., 2021). Given the harmful effects of behavioral problems, factors that help to reduce their detrimental effects should be identified and used to build effective intervention strategies.

Behavioral problems are often associated with environmental factors such as family and school settings (Joseph et al., 2021). Evidence is accumulating that daily stress (i.e., school- and family-related stress) may disrupt children’s lives. For instance, parental conflict can be stressful for most children (Liu and Wang, 2015). Labella and Masten (2018) found parental conflict to be associated with more behavioral difficulties. Moreover, in a longitudinal study, Westrupp et al. (2018) revealed that repeated early-life exposure to parental conflict consistently predicted children’s behavioral problems at 10–11 years old. Researchers have also identified school-linked stress, which may contribute to the emergence of behavioral problems. For example, involvement in school bullying is a major cause of stress for children (Swearer and Hymel, 2015). Children who experience bullying manifest emotional and behavioral difficulties (Garaigordobil and Machimbarrena, 2019). Additionally, Collins et al. (2017) indicated that children with high (versus low) teacher-child conflictual relationships exhibited more behavior problems in middle childhood. The literature therefore suggests that daily stress may increase the risk of behavioral problems.

In the stress-coping model (Folkman and Lazarus, 1988), cognitive appraisal, as well as coping resources and strategies, are the main factors that help individuals, and which explain and predict adjustment outcomes. According to the model, during stressful events, individuals evaluate the demands of stressors and available coping resources, determine stressors’ potential impact (i.e., establish whether they pose a threat), and identify potential coping strategies. Based on this model, children may interpret chronic difficulties as stressful events, identify available coping resources (e.g., social support), and determine how to use them to reduce the negative effects of stressors. Social support acts both as a protective factor against depression and plays a buffering role in the relationship between daily stress and depression (Ouyang et al., 2020). Therefore, social support may offer protection from the negative impact of stress.

The family unit is central to children’s growth. Parents are responsible for instilling their values, attitudes, beliefs, and behavior in their children, providing a framework for children to develop the ability to behave adaptively in later years (Blinkhorn et al., 2001). Family functioning, which embodies characteristics of the family system, has drawn increasing attention from psychologists and family researchers (Caprì et al., 2019; Pérez-Fuentes et al., 2019). For the current study, we focused on family functioning—which refers to the social and structural properties of the global family environment, encompassing communication, affective involvement, problem-solving, values and norms, and family roles—as it provides a broader perspective than the examination of parenting styles or parental behavior modeling alone (Mobach et al., 2020).

The process model of stress and coping (Armstrong et al., 2005) provides a theoretical framework to explain how families function; it emphasizes that coping resources and the use of coping strategies moderate vulnerability to the effects of stress. Within the domain of coping resources, the model acknowledges the contribution of factors such as specific aspects of family functioning. The protective-support hypothesis also posits that the companionship, status, and sense of purpose provided by family life buffers against chronic stressors in everyday life (Schwab et al., 2006). Functional families may help to prevent problematic behaviors in children by enhancing their cognitive restructuring, encouraging them to consider stressors as less threatening, and providing them with appropriate feedback and behavioral models (Markiewicz et al., 2001).

Empirical research has also revealed that good family functioning or cohesion acts as a protective factor to promote resilience. For example, family functioning is negatively associated with externalizing problems (Richmond and Stocker, 2006; Li et al., 2018; Pérez-Fuentes et al., 2019). Good family functioning is characterized by open communication, high levels of support, the expression of feelings and thoughts, and cohesion among family members (Delalibera et al., 2015). Tichon and Shapiro (2003) demonstrated that providing material assistance, offering emotional support, and aiding social interaction could protect children against the adverse effects of stress. Moreover, a 2-year longitudinal study indicated that healthy family functioning exhibited a significant buffering effect on subsequent aggression following exposure to violence (Deane et al., 2018). Cultural comparative research suggests that cultural background, in terms of individualism-collectivism, influences the impact of family support on individual-level outcomes (Stock et al., 2016). Unlike individualistic cultures that endorse self-reliance, personal freedom, and independence, collectivistic cultures deeply value in-group codependence (Wang et al., 2019; Wang et al., 2021). China is a traditional collectivistic culture that values strong family ties and frequent contact (Hofstede, 2001). Thus, in Chinese culture, family plays an important role in helping children to cope with daily stress.

According to the process model of stress and coping (Armstrong et al., 2005), protective factors (in a given context) can buffer against the negative impact of stress on students’ adjustment. In addition to family functioning, the classroom environment is a protective factor (Dilalla and Mullineaux, 2008). The classroom is a basic unit where Chinese primary school students study, play, socialize, and grow up with a stable group of classmates and several teachers across 6 years of schooling (Wang et al., 2018). The classroom environment—which encompasses a broad range of educational concepts, such as the psychological environment created through social contexts, teacher characteristics and behaviors, peer relationships, and discipline—strongly influences student outcomes, particularly in China’s school system (Jiang, 2004; Chen et al., 2006). Collective cultures deeply value interdependent ties among individuals, group loyalty, conformity to collective standards, and respect for authority (Romi et al., 2009).

One study of 1,941 pairs of monozygotic twins showed that twins in the same classrooms were more similar in terms of behavioral problems than twins placed in different classrooms, indicating that the classroom environment affects children’s behaviors (Dilalla and Mullineaux, 2008). Previous research has also shown that some aspects of the classroom environment play a vital role in children’s behavioral problems. For instance, teachers are one of the most significant adults in children’s lives as they provide comfort, guidance, and support to children (Marengo et al., 2021). In addition, peers provide students with companionship, assistance, a sense of belonging, and enjoyment at school (Gowing, 2019). Research has indicated that conflictual teacher-child relationships and problems in peer relationships could trigger various behavioral problems (Zhu et al., 2016). In a longitudinal study, Kim and Nho (2017) showed that peer relationship difficulties significantly predicted subsequent aggressive behaviors, even after controlling for previous degrees of aggression. Moreover, Jia et al. (2018) found that harmonious teacher-student bonds ameliorated the adverse impact of peer victimization on psychological security, which resulted in less internet addiction behavior among Chinese children. Abou-ezzeddine et al. (2007) also found that positive peer relationships could enhance children’s positive perceptions of the school climate, and in turn mitigate the adverse impact of environmental risk factors on aggressive behavior, especially in a collectivist culture like that of China. The literature reviewed above indicates that a positive classroom environment may buffer against behavioral problems in children who experience daily stress.

Although relatively few studies have investigated the moderating effects of family functioning and the classroom environment on the association between daily stress and behavioral problems, according to all the aforementioned theories and literature jointly, we can infer that healthy family functioning and a favorable classroom environment may alleviate the relationship between daily stress and behavioral problems.

This study aimed to identify environmental factors that could protect children against the negative effects of daily stress. We examined the relationship between daily stress and behavioral problems in children, and explored the moderating effects of family functioning and the classroom environment on this relationship. Specifically, we used the latent moderated structural (LMS) equation method to test three hypotheses:

*H1: Daily stress positively predicts children’s behavioral problems.*

*H2: Family functioning moderates the relationship between daily stress and behavioral problems in children.*

*H3: The classroom environment moderates the relationship between daily stress and children’s behavioral problems.*

## Materials and Methods

### Participants and Procedure

The participants were 1,495 children and their parents or guardians. We recruited the children from five randomly selected elementary schools in Southwest China. Of the 1,495 participants, 1,450 (ages 9–13, *M* = 10.91, *SD* = 0.96 years) completed the survey for a 96.99% response rate. The final sample of children consisted of 749 (51.7%) boys, 692 (48.6%) girls, and nine with their gender not reported. Among them, 446 (30.8%), 481 (33.2%), and 523 (36.1%) were in the fourth, fifth, and sixth grades, respectively. Class size varied from 46 to 69 students, with a typical number of approximately 52. The final sample of parents and guardians consisted of 683 (47.1%) fathers, 735 (50.7%) mothers, 21 (1.4%) other types of guardians (e.g., grandparents), and 11 (0.8%) with guardian information not reported. The participants were all of the Han ethnic background.

The Research Ethics Committee for psychological research at the authors’ institution approved this study (ID: LL2021018). Before administering the surveys, participating schools provided parents with an explanation regarding the study and assurance that participation was voluntary and that data would remain confidential. Informed consent was obtained from all participants. The children completed the three questionnaires (daily stress, family functioning, and the classroom environment) in their classrooms during regular school hours, with guidance provided by trained graduate researchers. Subsequently, the children delivered a questionnaire on their behavioral problems to their guardians (one in each family) to fill out. Next, the completed questionnaire was placed in a sealed envelope and forwarded to the researchers.

### Measures

#### Daily Stress

Children’s daily stress was assessed using the Children’s Daily Stress Inventory, developed by “The Study of Chinese Children and Youth’s Psychological Development” project team (Dong and Lin, 2011). The inventory lists six events occurring in everyday interactions that could have negative effects on children’s development: bullying, examination failure, study load, financial problems in the family, family conflict, and chronic physical illness in a family member. Each item assesses the occurrence and self-reported impact of the event on a six-point Likert scale ranging from 1 (*no, this did not happen to me*) to 6 (*yes, extremely*). The total score is the sum of the scores of all items, with higher scores indicating higher levels of daily stress. The scale has demonstrated good reliability and validity in Chinese children (Yang et al., 2015). The Cronbach’s alpha for the scale was 0.78 in this study.

#### Behavioral Problems

Behavioral problems were assessed using the Chinese version of the Strengths and Difficulties Questionnaire (SDQ) (Du et al., 2006). It consists of 25 items divided equally between five subscales: conduct problems, hyperactivity-inattention, emotional symptoms, peer problems, and prosocial behaviors. Scores for all items in the first four subscales were summed to generate a total difficulty score, which represented the extent of children’s overall behavioral problems. Responses used a three-point scale ranging from 0 (*not true*) to 2 (*certainly true*). Because parents and guardians are a primary source of information on everyday interactions with children, the parental version of the SDQ (Du et al., 2006) was administered to parents (one in each family). The scale has been widely used in Chinese children and demonstrated excellent psychometric properties (Ren et al., 2018). The Cronbach’s alpha coefficients for the subscales ranged from 0.69 to 0.85, and for the total scale was 0.73.

#### Family Functioning

Family functioning was evaluated with the Family functioning Assessment Scale, developed by “The Study of Chinese Children and Youth’s Psychological Development” project team (Dong and Lin, 2011). The scale consists of six self-reported items, which evaluate perceived family cohesion, or the degree of commitment and help family members provide for one another (e.g., “When we face a problem, our family members can solve the problem together and count on each other”). Responses used a five-point Likert scale ranging from 1 (*not at all characteristic or true of me*) to 5 (*extremely characteristic or true of me*). Item scores were summed to provide a total score. The Family functioning Assessment Scale has demonstrated good reliability and validity in Chinese children (Shi et al., 2017; Li et al., 2018). In the present study, Cronbach’s alpha for the total scale was 0.82.

#### Classroom Environment

The children’s perception of their classroom environment was assessed using the revised version (Dong and Lin, 2011) of the Classroom Environment Scale (Jiang, 2004). The scale includes 24 items examining the following five dimensions: Teacher-student relationships (e.g., “Our head teacher is accessible to students”), peer relationships (e.g., “The students in our class get on very well”), class order and discipline (e.g., “When the teacher delivers a lesson, students keep quiet”), competitive atmosphere (e.g., “Each student seems eager for superiority over others”), and learning burden (e.g., “We rarely have time to play and relax”). Responses used a five-point scale ranging from 1 (*not at all true for my class*) to 5 (*very true for my class*). The Classroom Environment Scale has proven reliable and valid in previous studies (Ren et al., 2011; Bai and Jin, 2016). In the present study, the Cronbach’s alpha coefficients ranged from 0.71 to 0.87.

### Statistical Analysis

Since our data spanned two levels of analysis, with individual perceptions of the classroom environment being nested within their classrooms, we initially used hierarchical linear multilevel (HLM) modeling, which explicitly accounts for nested data. However, the intraclass correlation, representing the proportion of observed variance of a variable between peer groups, was only 0.03. This means that only 3% of the observed variance was between groups. Given the lower intraclass correlation coefficient value, we ultimately did not apply HLM modeling (Luke, 2004). Instead, we used the LMS equation approach, a new method developed to examine general interaction models with latent interaction effects. Hence, we performed all analyses based on the LMS equation approach using the software Mplus, version 7.4 (Muthén and Asparouhov, 2015, Los Angeles, CA, United States). However, this method has several limitations: (1) traditional model fit indices used in structural equation modeling (SEM) are not provided for LMS models; and (2) information regarding the proportion of variance explained by latent interactions is not available in Mplus. As such, interaction effects are difficult to interpret using only the standard output. Thus, following previous studies using LMS models, we employed a two-step method to assess each LMS model’s overall fit (Maslowsky et al., 2015).

We obtained comparative fit index (CFI), Tucker-Lewis Index (TLI), root-mean-square error of approximation (RMSEA), and chi-square (χ^2^) values from Model 0. Using a log-likelihood ratio test, denoted as *D*, we compared the relative fit of Model 0 (the null model, whereby the interaction is not estimated and therefore assumed to be zero) and Model 1 (the alternative model, whereby the interaction is estimated) using the following equation:


D=-2⁢[(log-likelihood⁢for⁢Model0)-(log-likelihood⁢for⁢Model⁢1)]

To analyze the dependent variable (i.e., children’s behavioral problems), we derived the total variance explained in Models 0 and 1, *R*_*Y*__0_^2^ and *R*_*Y*__1_^2^, respectively, from the Mplus standardized output. Finally, Δ*R*_*Y*_^2^ = *R*_*Y*__1_^2^ − *R*_*Y*__0_^2^, the difference between these two *R*^2^ values, provided the proportion of *R*^2^ explained by the interaction.

## Results

### Preliminary Analyses

Table 1 shows descriptive statistics and correlations. The analysis of variance (ANOVA) results indicate a significant main effect of grade, *F*(2,1447) = 8.14, *p* < 0.001, η_*p*_^2^ = 0.01. Parents reported significantly lower scores for sixth-graders than for fourth- and fifth-graders on behavioral problems. The outcomes of the independent-samples *t* test point to a significant difference in behavioral problems according to sex, *t*(1448) = 2.18, *p* < 0.05, *d* = 0.11. Parents rated boys (*M* = 11.24, *SD* = 5.41) significantly higher than girls (*M* = 10.64, *SD* = 5.10). Given these substantial differences in behavioral problems based on grade and sex, we included these variables as covariates in subsequent moderator analyses.

**TABLE 1:** Means, *SD*s, and correlations among study variables (*N* = 1,450).

	*M*	*SD*	1	2	3	4	5	6	7
1. DS	11.03	4.80							
2. FF	24.75	4.86	−0.37**						
3. CE	17.50	2.05	−0.29**	0.47**					
4. CP	1.78	1.52	0.19**	−0.20**	−0.11**				
5. H-I	4.09	2.44	0.20**	−0.20**	−0.14**	0.46**			
6. ES	2.42	2.01	0.22**	−0.15**	−0.06**	0.39**	0.28**		
7. PP	2.61	1.66	0.16**	−0.16**	−0.07**	0.18**	0.12**	0.35**	
8. BP	10.90	5.27	0.28**	−0.26**	−0.16**	0.71**	0.74**	0.73**	0.55**

SD: standard deviation; DS = daily stress; FF = family functioning; CE = classroom environment; CP = conduct problems; H-I = hyperactivity-inattention; ES = emotional symptoms; PP = peer problems; BP = behavioral problems. **p < 0.01.

### Measurement Model

We performed confirmatory factor analysis (CFA) to test the measurement model, which comprised the four latent variables: (1) daily stress; (2) behavioral problems; (3) family functioning; and (4) the classroom environment. The latent daily stress variable was indicated by six items. The latent behavioral problems variable was denoted by problems with conduct, hyperactivity-inattention, emotional symptoms, and peer problems. The family functioning latent variable was represented by six items. The classroom environment latent variable was embodied by teacher-student relationships, peer relationships, class order and discipline, a competitive atmosphere, and learning burden. The overall model yielded an acceptable fit, χ^2^(183) = 907.66, *p* < 0.001, RMSEA = 0.05, CFI = 0.92, TLI = 0.91. Standardized factor loadings ranged from 0.52 to 0.84 and were significant at *p* < 0.001.

### Moderator Analyses

**FIGURE 1:**
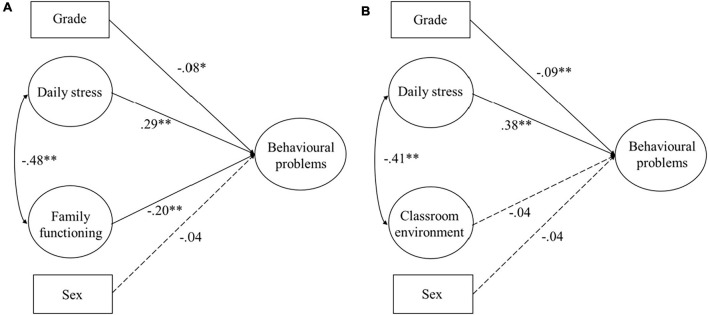
Structural models without a latent variable interaction. **(A)** Model 0: Main effects of daily stress and family functioning; **(B)** Model 0: Main effects of daily stress and classroom environment. *p < 0.05, **p < 0.01.

Model fit indices were calculated. Model 0 (Figures 1A,B) was estimated. Both models (Figures 1A,B) showed acceptable fit to the data: Model 0 (Figure 1A): χ^2^(131) = 527.20, *p* < 0.001, RMSEA = 0.04, CFI = 0.91, TLI = 0.90; and Model 0 (Figure 1B): χ^2^(115) = 326.80, *p* < 0.001, RMSEA = 0.05, CFI = 0.90, TLI = 0.89. Daily stress positively predicted the children’s behavioral problems (β = 0.29, *t* = 5.76, *p* < 0.001 and β = 0.38, *t* = 8.78, *p* < 0.001), thus supporting Hypothesis 1. In addition, family functioning significantly predicted behavioral problems (β = −0.20, *t* = 4.27, *p* < 0.001), but the classroom environment did not (β = −0.04, *t* = 1.13, *p* > 0.05).

**FIGURE 2:**
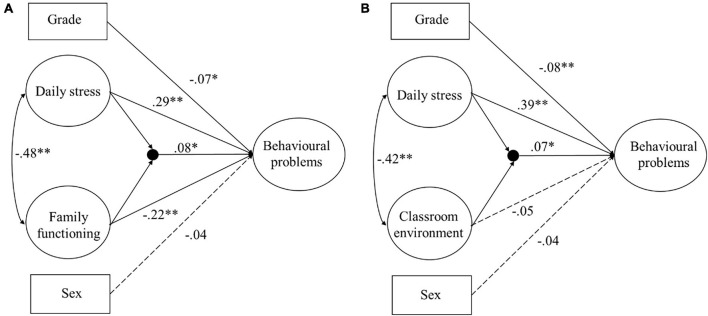
Structural models with the latent variable interaction. **(A)** Model 1: Model including the latent variable daily stress × family functioning interaction, depicted as a filled circle according to Mplus standard notation; **(B)** Model 1: Model including the latent variable daily stress × classroom environment interaction. *p < 0.05, **p < 0.01.

The two Models (Figures 2A,B) were then estimated. The fit of Model 1, relative to Model 0, was determined *via* a log-likelihood ratio test to compare the two models’ log-likelihood values; the log-likelihood difference values for Models 0 and 1 were *D* = 5.19 and *D* = 4.30, respectively. The chi-square distribution shows that the log-likelihood ratio test was statistically significant (*p* < 0.05), indicating that Model 0 (the model without the interaction) exhibited a significant loss in fitness relative to Model 1 (the model with the interaction). The interaction effects of both daily stress × family functioning (β = 0.08, *t* = 2.16, *p* < 0.05) and daily stress × the classroom environment (β = 0.07, *t* = 2.06, *p* < 0.05) were statistically significant after controlling for grade and sex; therefore, Hypotheses 2 and 3 were supported.

The method described above was used to interpret the interaction effect size. The daily stress × family functioning and daily stress × the classroom environment interactions explain 2.6% and 1.7% of the variance in the children’s behavioral problems, respectively.

**FIGURE 3:**
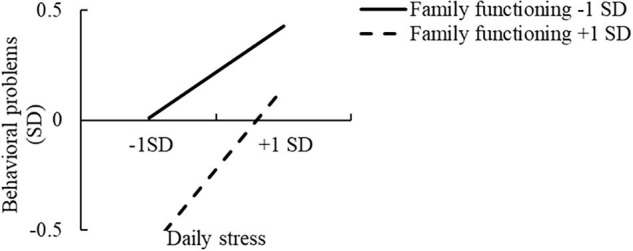
Interaction between daily stress and family functioning, predicting children’s behavioral problems.

**FIGURE 4:**
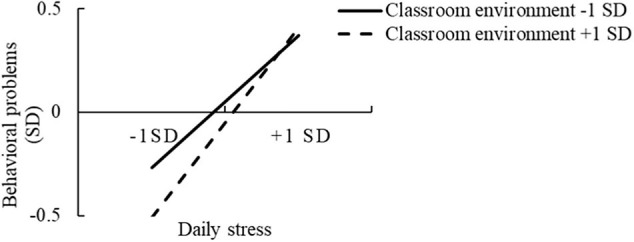
Interaction between daily stress and classroom environment, predicting children’s behavioral problems.

Regarding the underlying processes, a simple slope analysis (Aiken and West, 1991) revealed that the positive relationships for both high family functioning and high classroom environment were significantly different from zero, *simple slope* = 1.47, *t* = 7.32, *p* < 0.001, and *simple slope* = 1.62, *t* = 7.75, *p* < 0.001, respectively. Moreover, the positive relationships for both low family functioning and low classroom environment were significantly different from zero, *simple slope* = 0.81, *t* = 4.52, *p* < 0.001, and *simple slope* = 1.11, *t* = 5.89, *p* < 0.001, respectively. We plotted the interactions (Figures 3, 4) to aid interpretation, which suggest that both family functioning and the classroom environment exerted stronger protective influences on behavioral problems when daily stress level was lower. Note that children with favorable (versus poor) family functioning and classroom environments exhibited fewer behavioral problems, regardless of the level of daily stress.

## Discussion

We examined whether family functioning and the classroom environment could function as moderators between daily stress and behavioral problems. We sought to overcome the limitations of previous research in this field by investigating the effects of daily stress on children’s behavioral problems in the context of broader family circumstances and classroom environments, focusing on the moderating processes within Chinese culture.

We explored the effects of demographic factors on behavioral problems. Regarding grade differences, in line with other Chinese studies (Wu et al., 2015; Wu et al., 2017), children in grade 6 reported the lowest levels of behavioral problems compared to other grades. This finding may be attributable to children’s acquisition of self-control, which develops over time (Duckworth and Gross, 2014; Zhi et al., 2020); both cross-sectional and longitudinal studies suggest that self-control in childhood strongly predicts individual behavioral problems (Daly and Perez, 2009; Schlam et al., 2013). Thus, behavioral problems showed a downward trend with grade in the present study. Regarding sex differences, consistent with previous findings (Wu et al., 2015; Cui et al., 2021), boys tended to have higher scores than girls on behavioral problems, as reported by parents. The peer-socialization model may provide an interpretation of this outcome, which implies that children are more likely to assimilate the characteristics of same-sex groups, placing boys at risk for behavioral problems (Rose and Rudolph, 2006).

### Daily Stress and Behavioral Problems

One of our goals was to provide data on the relationship between daily stress and behavioral problems in Chinese children. After controlling for sex and grade, children reporting higher daily stress exhibited more parent-reported behavioral problems, which is in accordance with previous research indicating that daily stressors have a cumulative effect on children, and distress associated with these stressors is related to various behavioral difficulties (Garaigordobil and Machimbarrena, 2019; Zhang and Mersky, 2020). For instance, Escobar et al. (2011) found that daily stress is a significant predictor of emotional and behavioral maladjustment in Caucasian schoolchildren. Roberts et al. (2018) also found that the amount of daily stressors appears to be strong predictors of psychosocial outcomes and risk behaviors among children in the United States. In a Chinese study, Chen and Hong (2010) also examined the relationship between daily stressors and behavioral problems among children in fifth and sixth grade, and found that daily stressors directly predicted both internalizing and externalizing problems.

The daily stressors included in our study were children’s daily experiences. Adults may regard these things as trivial or as part of life that children must learn to live with; however, such stressors may become sources of pressure for elementary school children, as children’s social circles are mostly restricted to their home and school, and their problem-solving abilities and resources are limited. Moreover, the pressure is likely to be exacerbated by the recurrent nature of daily stressors (Chan et al., 2016). Compas (2006) postulated that chronic stress impacts health through its allostatic load. The organism functions within a level of normal resistance. However, when stress levels exceed adaptive resources, they will pose threats to homeostasis; this may lead to high-cost responses, which include elevations in psychosomatic reactions and the elicitation of harmful behaviors (i.e., aggression, risk-taking, and self-damaging conduct; Rice, 2012). In addition, daily stress in childhood can disrupt the neural systems responsible for stress responses, predict emotional symptoms, and impair an individual’s ability to cope with social interactions and potential threats, potentially leading to behavioral problems (Shonkoff et al., 2012). Chinese research has shown that daily stress related to academic performance, schoolwork, and relationships with parents, teachers and peers is associated with anxiety (Chan et al., 2015).

### The Buffering Role of Family Functioning

The results show that family functioning served as a buffer in the relationship between daily stress and behavioral problems; this provides empirical evidence for the stress-coping model, which states that the ways in which children deal with stress affect mental health (Lazarus and Folkman, 1984). The results also support the protective-support hypothesis whereby social support, as a coping resource, can reduce the adverse mental health risks of stress (Schwab et al., 2006).

In terms of family functioning, children at this age rate their parents as their most frequent providers of social support in times of stress, and healthy family functioning can provide a supportive context for children’s physical, psychological, and social growth, and promote a wide range of experiences to avoid emotional suppression through open communication, which in turn decreases behavioral problems (Thompson et al., 1992). The societal culture where a family exists contributes to family functioning. Collectivism is a significant value practiced by Chinese society. Values such as cooperation, helpfulness, obedience, interdependence, and maintaining harmonious interpersonal ties are encouraged, especially during childhood (Kling, 1995); these relationships help children face challenges and obstacles (Sumari et al., 2019). Moreover, they can buffer against the effects of disadvantages and offer significant psychological resources for healthy development. Healthy family functioning is connected to children’s ability to deal with daily life and unforeseen circumstances (Sumari et al., 2019). For example, a recent study from China suggests that good family functioning fosters better coping with life events and helps to relieve psychological problems, such as anxiety and loneliness (Pan et al., 2021). Thus, compared to children with poor family functioning, children with good family functioning can seek formal and informal advice or emotional assistance from family members to positively handle daily stress; they hence exhibit fewer behavioral problems.

### The Buffering Role of the Classroom Environment

The finding that the classroom environment serves as a buffer in the relationship between daily stress and behavioral problems is another novel contribution to extend the literature, which also provides empirical evidence for the stress-coping model (Lazarus and Folkman, 1984) and the protective-support hypothesis (Schwab et al., 2006).

Differences exist in the perception of the classroom environment and its effects on students’ development between Western and Chinese regions (Wong et al., 2016). In China, students have a fixed classroom, with a specially designated classroom teacher before college. Educators regard the class as a collective or social system. Close teacher-student bonds and serious classroom discipline are two salient features of the classroom environment; thus, a typical classroom in China often has good classroom management and obedient and attentive students (Wang et al., 2017). Empirical research has repeatedly shown that the quality of the classroom environment is linked to behavioral outcomes in primary and secondary schools. For instance, Xie (2000) found that students in classes with high (versus low) perceived rule clarity, teacher support, and student involvement reported greater contentment and fewer absences, especially those from collectivist backgrounds. In addition, at-risk students can benefit from a positive classroom environment. The classroom environment and its dimensions can moderate the influence of risk factors (e.g., socioeconomic status and low self-efficacy) on students’ outcomes (e.g., academic achievement) (Malecki and Demaray, 2006; Martin and Rimm-Kaufman, 2015). Thus, a positive classroom environment can serve as a protective factor against the harmful impact of daily stress on behavioral problems in China.

Expanding on previous findings (Zhu et al., 2016; Li et al., 2018), the results revealed that in the context of low levels of daily stress, children with good family functioning and a favorable classroom environment exhibited fewer behavioral problems than those with poor family functioning and an unfavorable classroom environment; whereas in the context of high levels of daily stress, children with both good and poor family functioning and classroom environment showed more behavioral problems. Regarding the moderating processes, the stress-vulnerability hypothesis offers an appropriate framework for grasping the underlying processes observed in our results; this theory argues that possessing certain attributes is generally advantageous, particularly when stress levels are low. However, these protective factors might lose their ability to counteract risk once it reaches a certain level. Hence, it might be difficult for individuals exposed to severe adversity to achieve positive outcomes (Vanderbilt-Adriance and Shaw, 2008; Li et al., 2012). In line with the stress-vulnerability hypothesis, the findings suggest that healthy family functioning and a positive classroom environment might not be sufficient to protect children from behavioral problems when facing high levels of daily stress.

### Implications

The present study contributes to the literature regarding children’s daily stress and behavioral problems by extending the prior focus on major life events in examining daily stress among Chinese children. Moreover, the current study showed that the beneficial effects of good family functioning and a favorable classroom environment may be overwhelmed by high levels of daily stress; this offers empirical support for the stress-vulnerability hypothesis.

In practice, first, we should be alert to the negative effect of daily stress on children’s behavioral problems. Second, the buffering effects of family functioning and the classroom environment are more likely to be observed in children who report minor stress. These findings could serve to warn educators and caregivers that they should pay attention to the creation of functional families and favorable classroom environments. However, we should not exaggerate the roles of good family functioning and a favorable classroom environment. When children are exposed to severe daily stressors, the potential values of functional families and favorable classroom environments in interventions to reduce behavioral problems are limited. This may be because other family factors (i.e., parental stress), personality (i.e., extraversion, trait resilience), and mindfulness training also moderate the relationship between daily stress and behavioral problems (Korotkov, 2008; van de Weijer-Bergsma et al., 2014; Weiss et al., 2015). Therefore, to reduce children’s behavioral problems, educators and caregivers need to help families stay strong and build healthy classroom environments on the one hand, and assist children in reducing their stress levels.

### Limitations and Future Directions

While the results provide new insight into how environmental factors reduce children’s stress-related behavioral problems, the study has some limitations. First, to gage stressors, we used retrospective and subjective evaluations, which could have been biased. Future research should include alternative measures (e.g., daily diaries) to minimize such problems by focusing on objective, external measurements and allowing participants to report on their experiences immediately after they occur. Second, our study was limited to a restricted population of children from Southwest China. Thus, some caution should be exercised in drawing conclusion, and future studies should use a larger, more diverse sample to ensure the generalizability of the findings. Third, since we focused selectively on environmental domains as the sole explanatory factor, future research should analyze additional moderating mechanisms across multiple domains. Other internal resources, such as self-esteem or trait resilience, may also be determinants in the buffering process. Fourth, although we found that family functioning and the classroom environment can buffer against the effect of daily stress on behavioral problems in collectivist cultures such as that of China, whether there are differences in the stress-buffer effects among different cultures is unclear. Hence, cultural comparative research is needed. Last, our study is based on a cross-sectional design, which does not allow us to make definitive conclusion that daily stress leads to more behavioral problems. To better ascertain causality, longitudinal studies are needed to verify the current findings.

## Conclusion

The present study provides new, useful information about the association between daily stress and behavioral problems and the underlying psychological mechanisms. The findings imply that daily stress is positively related to behavioral problems, and that both family functioning and the classroom environment moderate the relationship between daily stress and behavioral problems after controlling for sex and grade. Moreover, a functional family and a favorable classroom environment are generally advantageous, particularly when the daily stress level is low. These results provide novel information, emphasizing the importance of a functional family and a favorable classroom environment as additional factors that may protect against behavioral problems in children who experience mild daily stressors.

## Data Availability Statement

The raw data supporting the conclusions of this article will be made available by the authors, without undue reservation.

## Ethics Statement

The studies involving human participants were reviewed and approved by the Research Ethical Committee for psychological research of Liaoning Normal University. Written informed consent to participate in this study was provided by the participants’ legal guardian/next of kin.

## Author Contributions

LW conceived the study, participated in its design, and drafted the manuscript. FD performed the statistical analysis and contributed to the design of the study and the interpretation of data. TH assisted with literature review, proofed the results, and participated in manuscript drafting and revision. GC participated in the design of the study, coordinated the work, and carefully revised the manuscript. XC contributed to editing the manuscript. All authors agreed with the final manuscript.

## Conflict of Interest

The authors declare that the research was conducted in the absence of any commercial or financial relationships that could be construed as a potential conflict of interest.

## Publisher’s Note

All claims expressed in this article are solely those of the authors and do not necessarily represent those of their affiliated organizations, or those of the publisher, the editors and the reviewers. Any product that may be evaluated in this article, or claim that may be made by its manufacturer, is not guaranteed or endorsed by the publisher.
